# Flaxseed Oilcake: An Ingredient with High Nutritional Value in the Realization of Innovative Food Products

**DOI:** 10.3390/foods14071087

**Published:** 2025-03-21

**Authors:** Ancuța Petraru, Sonia Amariei, Lăcrimioara Senila

**Affiliations:** 1 Faculty of Food Engineering, Stefan cel Mare University, 720229 Suceava, Romania; sonia@usm.ro; 2 National Institute for Research and Development of Optoelectronics INOE 2000, Research Institute for Analytical Instrumentation, 67 Donath Street, 400293 Cluj-Napoca, Romania; lacri.senila@icia.ro

**Keywords:** flaxseed oilcake, polyphenols, antioxidants, proteins, fibers

## Abstract

The by-products of the oil cold pressing of flaxseed are deemed to be safe, edible products. They have been shown to possess high nutritional value (compared with the seeds, they are richer in proteins and minerals) and adequate functional parameters (i.e., a high water-holding capacity and emulsion stability). In oilcakes, we found a portion of oil that was richer in unsaturated fatty acids (87.90%) than flax seeds (57.40%). Mg predominates in flax seeds, while Ce is predominant in flaxseed oilcake. Regarding essential amino acids, the seeds (76.71%) were found to be richer than the oilcake (70.46%). The use of methanol, low extraction temperatures, s high ultrasonic amplitude, and longer times resulted in the highest antioxidant capacity and phenolic content for flaxseed oilcake. Our analyses showed that oilcakes can be utilized as a functional ingredient or for the extraction of bioactive compounds, which can be incorporated into food products due to their nutritional, social, and economic benefits.

## 1. Introduction

The processing of agricultural products produces by-products and waste (998 million tons annually, of which more than 80% is organic waste.) [1,2]. The processing and manufacturing of oil crops generates 36% of the total amount of waste and by-products [3]. The management of these materials poses an inevitable problem because environmental protection (related to climate change) and sustainability must be taken into account [4,5]. A fundamental objective of the food industry is to minimize waste. The reevaluation of waste as a renewable resource is a pivotal step towards achieving this objective. This reevaluation will facilitate the creation of new products, aligning with the latest guidelines of the European Union regarding the circular economy [6,7]. In the literature, the most common practices used to valorize waste include their disposal into landfill or use as livestock feed [8,9].

Flaxseed (*Linum usitatissimum* L.), belongs to the *Linaceae* family. It is a major and ancient oil-bearing plant, which is used in culinary and industrial applications [10]. The growing consumer demand for nutritional foods that provide significant nutritional value, the prevention of diet-related diseases, and the enhancement of consumer wellness have prompted significant interest in incorporating flaxseed as a dietary ingredient [11,12].

While flaxseed boasts a multitude of health and nutritional benefits, its composition also features high concentrations of toxic compounds, which have the potential to interfere with nutrient bioavailability and potentially aggravate preexisting health concerns [13]. The primary anti-nutritive factors identified in flaxseed include cyanogenic glycosides, phytic acid, linatine, and protease inhibitors [14,15]. The deactivation or elimination of these components can be achieved using various methods, including physical (boiling, soaking, heating, grinding or lyophilization), biological (germination or fermentation), chemical (maceration, microwave, or ultrasound extraction with alkaline, acidic and water-based solvents), and mechanical (extrusion or roasting) techniques [13,16].

Flaxseed oilcake refers to the solid residue that is left over after the removal of the oil from the flax seeds. Because of its numerous medicinal, functional, and nutritional properties, oilcake has attracted considerable interest [17]. Oilseed cakes are currently underutilized as animal feed, although they contain high concentrations of valuable components and are low-cost and sustainable resources [18].

For the global food industry, flaxseed oilcakes are gaining significance as a functional food. Functional foods comprise food ingredients with physiological benefits that may contribute to the prevention/cure of various diseases [19]. An investigation of the literature regarding the employment of flaxseed oilcake in the food sector highlighted its introduction into bread (reducing fermentation times), dairy beverages (increasing viscosity and antioxidant activity), and as an additive to stabilize emulsions [20,21,22,23,24].

The ongoing search for food ingredients that improve nutrition and human health has led to the need to systematically study all aspects of flaxseed oilcake, starting with the seeds and all the transformations and nutrient losses that occur during oil extraction, and including the functional and technological properties that are highly important in the development of new food formulas. The novelty of this research lies in its summary of all the nutritional characteristic of flaxseed oilcake that make it suitable for utilization in the food sector, with the aim of transforming common food products into healthier and more nutritious products.

## 2. Materials and Methods

### 2.1. Materials

The materials utilized in the present study consist of flax seeds and flaxseed oilcake, which were procured from a commercial oil factory (OLEOMET, Bucharest, Romania). The flaxseed oilcake was subjected to a grinding and sifting process (with Vibratory Sieve Shaker AS 200 basic, Retsch, Haan, Germany), yielding particles measuring less than 400 µm.

### 2.2. Physical Properties

**Figure 1:**
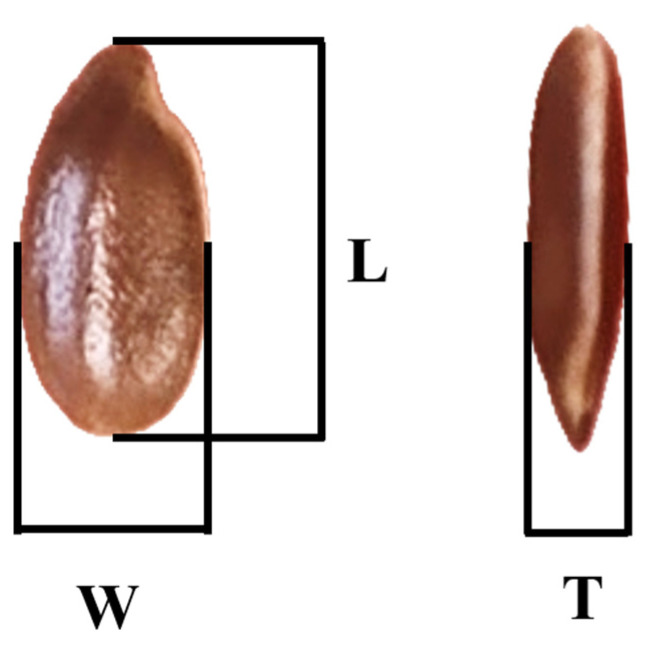
Dimensional representation of the seed.

A digital caliper (VOREL 15240, Toya, Wrocław, Poland) was used to measure the seeds with an accuracy of 0.003 mm. The dimensional parameters analyzed were width (W), length (L), and thickness (T) (Figure 1) [25].

The geometric parameters examined in this study (equivalent diameter—D_e_, surface area—S, projected area—A_p_, sphericity—ψ and volume—V) were calculated using the relationships presented below (Equations (1)–(5)) [26]:

(1) $$D_{e}\;(\mathrm{mm})=\sqrt[{3}]{W\times L\times T}$$

ψ = De/L(2)

(3) $$S\;({\mathrm{mm}}^{2})=\Pi \times {\mathrm{De}}^{2}$$

(4) $$A_{p}\;({\mathrm{mm}}^{2})=¾\;\times \;L\;\times \;W$$

(5) $$V({\mathrm{mm}}^{3})=W\;\times \;L\;\times \;T\;\times \;\Psi$$

Bulk density, true density, porosity, and mass were analyzed as gravimetric parameters. A group of whole seeds, randomized into five sets of 100 seeds each, was weighed (accuracy 0.1 mg) with the aid of an analytic balance (PARTEN AS 220.R2, Radwag, Bucharest, Romania).

Bulk density (p_b_) is defined as the seed mass divided by the total volume (Equation (6)). True density (p_t_) was analyzed using a pycnometer by measuring the mass of toluene displaced by the seeds (p = 0.867 g/mL) (Equation (7)). Porosity (ϕ) was calculated as the ratio of the previously determined densities (Equation (8)) [27]:

p_b_ (kg/m^3^) = M/V (6)

(7) $$p_{t}\;(\mathrm{kg}/m^{3})=(M_{s}\times p_{\mathrm{toluene}})/M_{T}$$

(8) $$\Phi \;(\%)=((p_{t}-p_{b})/p_{t})\;\times \;100$$

### 2.3. Nutritional Composition of Flax Seeds and Flaxseed Oilcake

A gravimetric method (ISO 665:2020, AOAC 935.29) [28,29] was employed to detect the water content in the samples. The drying process was carried out at 105 °C, util the mass of the materials remained constant [30].

Kjeldahl’s procedure (AOAC 950.48) [31] was used for protein determination. Briefly, in a Kjeldahl flask were placed 1 g of the sample, a Missouri catalyst, and 15 mL of sulfuric acid. The digestion was run for 2 h at 420 °C in order to obtain a clear blue solution. To perform the distillation, the solution was first diluted with water (50 mL er), and then 30 mL of 30% sodium hydroxide was added. Ammonium was collected in 25 mL of 4% boric acid. Quantification was performed by titrating with 0.1 N of hydrochloric acid using the Tashiro indicator. A control sample was prepared simultaneously [32,33].

In accordance with ISO 659:2009 and AOAC 920.39 [34,35], the quantification of the lipid content was performed with petroleum ether, employing an automatic Soxhlet extraction system [36].

Ash determination was performed by subjecting the sample to calcination at 550 °C until a constant mass was obtained (AOAC 923.03) [37,38]. A Megazyme assay kit (Megazyme, Wicklow, Ireland) was employed to assess the total content in dietary fiber in accordance with AOAC 985.29 [39].

The energy value and carbohydrate content were calculated using Equations (9) and (10), respectively [40]:

Carbohydrates (%) = 100 − (moisture + ash + protein + lipids + fibers)(9)

(10) Energy value (kcal/100 g)=9 × fat+4 × (protein+carbohydrates)+2 × fibers

### 2.4. Flaxseed Oilcake Characterization

#### 2.4.1. Safety Assessment

The safety of the flaxseed oilcake was established by determining the water activity index (AquaLab 4TE, Meter Group, Pullman, WA, USA), micotoxins–zearalenone, ochratoxinA, aflatoxin B1 and deoxynivalenol (ProGnosis Biotech, Larissa, Greece) and heavy metals (by ICP-MS, Agilent Technologies 7500 series, Santa Clara, CA, USA) [41,42].

#### 2.4.2. Functional Properties

The functional properties researched were the emulsion capacity and stability, foaming capacity and stability, water-holding capacity, oil-holding capacity, wettability and least-gelatinization concentration.

The measurement of bulk density was achieved via the introduction of 5 g of flaxseed oilcake into a 100 mL graduated cylinder. The cylinder was tapped lightly twenty times. Thereafter, the final volume was measured. Subsequently, the values were calculated as the fraction of the weight of the sample to its final volume [43].

The flaxseed oilcake water/oil uptake capacity (WHC/OHC) was determined by adding 1.00 g of flaxseed oilcake to 10 mL of distilled water or corn oil in a centrifuge tube. The tube was then kept at room temperature for a period of 30 min. This was followed by centrifugation for 20 min at 7000 rpm. The results were calculated using Equation (11) [44,45]:

WHC/OHC (g/g) = ((mass of sample after water absorption − mass of centrifuge tube) − mass of sample before analysis)/mass of sample before analysis(11)

The method proposed by Rani et al. [46] was used to analyze the emulsifying ability (EC). To that end, a suspension of 0.5 g of the sample and 100 mL of water was prepared and mixed for 20 min using a magnetic stirrer at 500 rpm. An emulsion was formed by mixing 30 mL of the resulting suspension with 10 mL of corn oil. To measure the final height, the emulsion was moved to a 50 mL graduated cylinder.

EC (%) = (height of oil layer/final emulsion layer) × 100(12)

The stability of the emulsion (ES) was determined according to Iyenagbe et al. [47]. This involved subjecting the emulsion to heating within the cylinder (30 min at 80 °C). Subsequently, the final height attained was recorded:

ES (%) = (emulsion height after heating/emulsion height before heating) × 100(13)

The foaming capacity (FC) and stability (FS) of the samples were determined according to the methodologies outlined by Naczk et al. [48]. To that end, a suspension was prepared by adding 3 g of the sample to 100 mL of water and was then mixed at 1600 rpm for 5 min. It was then moved to a 250 mL graduated cylinder for foam volume measurement.

FC (%) = (foam volume/initial suspension volume) × 100(14)

The stability of the foam was determined by monitoring its volume in time, every 10 min for 1 h (Equation (15)):

FC (%) = (foam volume after a period of time/initial foam volume) × 100(15)

The sample was dispersed in distilled water, forming concentrations ranging from 2% to 20%. Centrifuge tubes were heated for one hour in a boiling water bath and then rapidly cooled at 4 °C for three hours. The samples were then inverted, and the concentration that did not drop or slip was designated as least-gelatinized concentration [46,49]

Powder wettability was rated as poor, satisfactory, good, or excellent. This was based on how the powder behaved on the water surface immediately after being added [50].

#### 2.4.3. Color Parameters

The oilcake color was measured using a colorimeter (Konica Minolta CR-400, Tokyo, Japan) and interpreted using the CIELAB system. Lightness was measured according to the L* coordinate with values ranging from 0 to 100 for black to white. A negative value for the a* coordinate indicates a predominance of green color intensity, while a positive value indicates a predominance of red color intensity. Parameter b* ranges from −100, indicating the intensity of the blue color, to +100, denoting the intensity of the yellow color [51].

#### 2.4.4. FTIR-ATR (Fourier Transformed Infrared Analysis with Attenuated Total Reflectance)

A Nicolet iS20 spectrometer (Thermo Scientific, Karlsruhe, Germany) was employed to record the FTIR-ATR spectra in the region between 400 and 4000 cm^−1^. The conditions used were: 32 scans and a resolution of 4 cm^−1^. The spectra obtained were processed using the OMNIC program [42].

### 2.5. Fatty Acid Composition of Flax Seeds and Flaxseed Oilcake

For the analysis, the oils extracted from the samples (0.03 g) were mixed with isooctane (2 mL). Under vigorous stirring, a methanolic solution of 2 mol/L of potassium hydroxide was added (200 µL). The organic phase and sodium sulfide were blended and the subsequential supernatant was collected. Fatty acids were identified using a gas chromatograph with a flame ionization detector (Agilent Technologies, Wilmington, NC, USA). The capillary column used stationary polyethylene glycol and had a length of 30 m. The conditions used were confirmed using the procedure described by Petraru et al. [42]. The oven was initially set for 1 min at 60 °C, after which the temperature was increased to 200 °C (10 °C/min) for 2 min and to 220 °C (5 °C/min) for 20 min. The temperature for the injection port and the detector was set at 250 °C. The flow rates for air, helium, and hydrogen were set at 450, 30 and 40 mL/min, respectively. The fatty acids were detected and quantified in comparison with a standard mixture and the results were expressed as the relative level (%).

### 2.6. Amino Acid Composition of Flax Seeds and Flaxseed Oilcake

For the free amino acid analysis, 0.7 g of the sample was mixed with 6 mL trichloroacetic acid (15% TCA), the pH was adjusted to 2.2, and TCA was added to make 10 mL. The extracts were centrifuged for 5 min at 3000 rpm. This method was adapted from that of Dabadé et al. Therefore, the supernatants were filtered through a syringe filter (0.45 μm) and derivatized using a rapid amino acid analysis kit (Phenomenex Inc, Torrance, CA, USA). Amino acid analysis was performed using a gas-chromatograph with mass spectrometry (Shimadzu, Kyoto, Japan). The conditions used were in agreement with the procedure described by Chetrariu et al. [52].

### 2.7. Mineral Composition of Flax Seeds and Flaxseed Oilcake

Ash was dispersed in 0.73 mL of 65% nitric acid (Sigma-Aldrich, Darmstadt, Germany) and filled to 50 mL with demineralized water. The minerals (Li, V, Ce, Ni, Fe, Co, Zn, Cd, Ca, Cu, Mg, Cr, Mo, Tl, Mn, Ti, Sb, Se, Sr, Pb, Hg, Be and As) were estimated using an Agilent Technologies inductively coupled mass spectrometer (Santa Clara, CA, USA). The conditions used were the same as those used in the procedure described by Petraru et. al. [53]: the nebulizer was set at 0.9 mL/min, the carrier and completion gases were set at 0.92 L/min and 0.17 L/min, respectively, the mass range was 7–205 uma, and the acquisition and integration times were 0.1 s and 22.76 s, respectively.

### 2.8. Experimental Design for Determining Total Polyphenolic Content and Antioxidant Activity

#### 2.8.1. Extract Preparation

Preliminary analysis of the extant literature on the subject yielded four factors (temperature—A, time—B, amplitude—C, and solvent—D) that were subjected to variation (the coded values 1, 0 and −1 represent the maximum, mean and minimum levels of the variable range, respectively), with three replications predicted at the center point (Table 1). The extractions were carried out according to Table 1 (obtained using Design Expert (2024, trial version) and a Box–Behnken design) as follows: 1.00 g of defatted flaxseed oilcake was mixed with 20 mL of different solvents (methanol, water, and ethanol). The ultrasonic procedure was performed in an ultrasonic bath (Elma Transsonic TI-H15, Singen, Germany) at different times (10 min, 15 min, and 20 min), temperatures (30 °C, 40 °C, and 50 °C) and amplitudes (40%, 70%, and 100%). The frequency was kept at 45 Hz. The resulting extracts were centrifuged for 10 min at 2000 rpm. The supernatant was collected to investigate the antioxidant activity (AOA) and total phenolic content (TPC) of the flaxseed oilcake.

#### 2.8.2. TPC and AOA

To determine TPC, in 0.2 mL of the extract, we added (in order): 2 mL of Folin–Ciocâlteu reagent (diluted 1:10) and 1.8 mL of 7.5% sodium carbonate. The mixture was allowed to stand for 30 min at room temperature [54].

For the determination of AOA, 0.3 mL of the extract was mixed with 2.7 mL of 0.1 mM DPPH reagent (prepared in methanol). For 30 min, the blend was stored at an ambient temperature [55].

The reading of absorbances (UV-VIS-NIR spectrophotometer, Shimadzu Corporation, Kyoto, Japan) was executed at wavelengths of 750 nm for TPC and 517 for AOA. A gallic acid calibration curve was used to quantify the TPC (10–500 mg/L, R^2^ = 0.99658, y = 0.00484x + 0.16058).

### 2.9. Statistical Analysis

The resulting values were analyzed using Excel-Stat software (2024, trial version). To study the difference in nutritional components between the flax seeds and flaxseed oilcake, a Student’s *t*-test (*p* < 0.05) was applied. To determine the difference in amino acids, minerals, and fatty acids for the two samples, ANOVA with a Tukey test was used.

## 3. Results and Discussion

### 3.1. Physical Properties

It is important to determine the physical properties of flaxseed because of its considerable economic potential in the food and chemical industries. The physical properties provide useful data for the handling of the material after harvesting and for industrial processing [26].

The values obtained for the physical properties of flax seeds are: 2.24 mm, 4.78 mm, 1.05 mm, 0.48, 2.27 mm, 8.76 mm^2^, 16.15 mm^2^, 5.56 mm^3^, 38.97%, 605.19 kg/m^3^, 994.94 kg/m^3^, and 0.0070 g for width, length, thickness, sphericity, equivalent dimeter, projected area, surface area, volume, porosity, bulk density, true density, and mass, respectively (Table 2). These values are in line with the ranges found in the relevant literature for gravimetric properties, size, and shape (4.27–5.80 mm for length, 2.22–3.39 mm for width, 0.85–1.69 mm for thickness, 40.34–49.9% for sphericity, 11.68–20.25 mm^2^ for surface area, 7.93–8.60 mm^3^ for volume, 784.00–1340.00 Kg/m^3^ for true density and 545.00–726.60 Kg/m^3^ for bulk density) [25,26,27,56,57].

The physical properties can vary depending on the moisture content of the seeds. The seed size increases with moisture content, resulting in a decrease in the number of seeds occupying the same bulk volume [56].

Low sphericity indicates that the seed shape is flat and slips more easily on structural surfaces. This property is important in the design of dehulling and hopper equipment [26].

The surface area and porosity indicates how the seed will behave on vibrating surfaces during drying processing [58].

Bulk density is highly important because it determines the capacity of storage and transportation systems [46]. The true density of flaxseed is less than the density of water, indicating that the seeds float in water. This feature allows for the separation of the external materials [59]

As indicated in Table 3, among the correlations obtained for the shape parameters, only the correlation between length and width was found to be significant but moderate. The correlation between length and thickness was negative but weak, and the correlation between width and thickness was positive but also weak. Furthermore, it is notable that all correlations with mass proved to be positive yet relatively weak.

### 3.2. Nutritional Composition of Flax Seeds and Flaxseed Oilcake

Table 4 shows the nutritive evaluation of flax seeds and flaxseed oilcake. A comparison of the two samples revealed that the oilcake exhibited considerably higher concentrations of total protein (34.67%) and ash (4.79%) than the seeds (*p* < 0.05). Consequently, the seeds exhibited a higher energy value (354.87 kcal/100 g), which is attributable to their substantial oil content.

The lipid content decreased from 27.74% to 11.61%, while the total dietary fibers decreased from 26.10% to 24.90% once the seeds were subjected to cold oil extraction. The decrease in fibers is mainly due to the presence of more soluble than insoluble fibers.

A review of the extant literature reveals a divergence in the composition of flax seeds, with reported values ranging from 3.65% to 8% for moisture, 3.21% to 4.00% for ash, 36.41% to 46.02% for lipids, and 20.00% to 24.19% for protein [60,61,62]. By contrast, the nutritional composition of flaxseed oilcake (following cold extraction) has been examined in various studies, which have reported moisture content ranging from 6.89% to 9.27%, ash content ranging from 4.70% to 6.27%, protein content ranging from 14.40% to 41.97%, lipid content ranging from 6.11% to 21.4%, and total dietary fiber content ranging from 6.29% to 12.90% [18]. These variations could be the result of different varieties and the climatic conditions to which the plants are exposed, which would produce seeds with different levels of nutrients [63].

In terms of nutritional characteristics, the flaxseed oilcake meets consumer demands for high-protein, high-fiber, and low-fat foods that are obtained through sustainable practices and using minimal processing. The flaxseed oilcake should be considered for human consumption when there is an optimal balance between fat and protein. According to the accepted standards, for the human body, the ideal values for these nutrients are 3–5% and 20–25%, respectively [64]. The flaxseed oilcake fulfills the necessary criteria for protein.

### 3.3. Flaxseeed Oilcake Characterization: Safety, Functional Properties, and Qualitative Identification of Key Functional Groups

#### 3.3.1. Safety Assessment

According to the safety assessment (Table 5), the flaxseed oilcake does not contain heavy metals (lead, mercury, or cadmium) and all the micotoxins detected were in compliance with the legal European Union limits. The low water activity index (0.41 ± 0.001) of flaxseed oilcake prevents the growth of bacteria, molds, and yeasts [65].

#### 3.3.2. Functional Properties

Functional properties are important for the production, transportation, storage, and stability of food products. The water-holding capacity value (4.14 g/g) obtained in this study was in line with the values reported by others (1.99–6.90 g/g) [46,66,67,68,69,70]. The high value is presumably due to the sorbing and swelling of the mucilaginous polysaccharides in the hulls, or else due to the swelling of crude fibers and gelling of carbohydrates [60]. This property contributes to the enhancement of the stability, consistency, and viscosity of beverages [71]. It is also beneficial in improving the shelf life, quality and yield of food formulations (meat, bakery products, soups, and sauces) [72]. Oil-holding capacity is an important factor, as it improves the mouthfeel and flavor of the foods [73]. This property is preferred in meat products. The value found (1.19 g/g) is in range with those reported in the literature (0.90–3.13 g/g) [46,66,67,68,69,70,74,75].

Bulk density is important for storage capacity assessments [76]. This is the property that describes the weight of a powder and depends on the powder particles (shape, size, and compaction state) [42,53]. Others found values ranging from 0.21 to 0.80 g/mL [46,66,70].

An emulsion is a system with two liquid phases that are immiscible with each other (oil and water). The proteins and phospholipids are responsible for the formation and stabilization of emulsions. Their properties (quantitative content, flexibility, and hydrophobic character) can enhance molecular bonding at the oil–water interface, making the emulsions more stable [46,73]. These properties are useful in food applications such as infant formulae, baked goods, mayonnaise, ice cream, and salad dressings where emulsions are required to improve the mouthfeel and texture [17,77]. The values for emulsion capacity and stability in the literature vary between 33.60 and 80.60% and 72 and 128.46%, respectively [46,70,75].

The minimum concentration of flaxseed oilcake capable of forming a gel (converting a viscous fluid to a three-dimensional viscoelastic matrix via the ordered polymerization of molecules through heating) is 4% [78]. This concentration is much lower than the concentrations found in the literature [46,56,70,79]. The value found here is favorable for culinary applications because, by introducing small quantities, it is possible to produce gelled (custards and puddings) and thickened (soups, gravies, and sauces) products [77].

**Figure 2:**
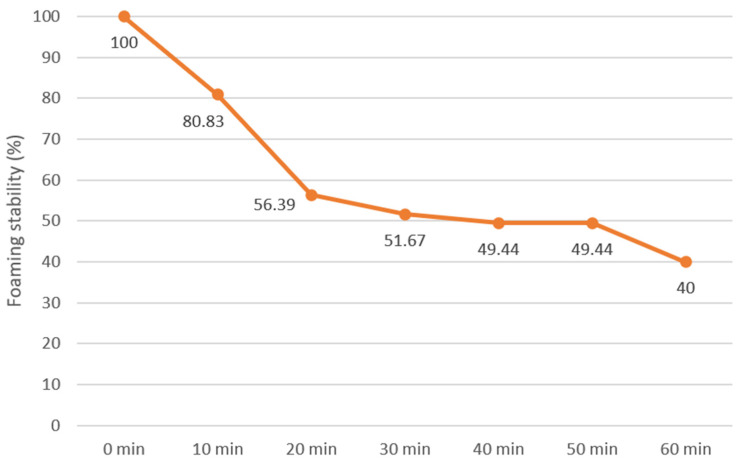
Stability of the foam over one hour.

The protein in flaxseed oilcake showed a low foaming (8.41%) capacity when subjected to whipping or beating. Additionally, the foam was not very stable. In an hour, the foam decreased from 80% to 40% (Figure 2). The low values found make it unsuitable for incorporating into meringues or whipped toppings where aeration is desired [17]. Other authors found values between 7.82% and 27.00% for foaming capacity, and its stability decreased from 82% to 22% within an hour [56,67,79].

The color parameters of the flaxseed oilcake are relatively dark (low luminosity value) and more yellowish than reddish in tone (positive data for a* and b*).

#### 3.3.3. Fourier Transform Infrared-Attenuated Total Reflection (FTIR-ATR)

**Figure 3:**
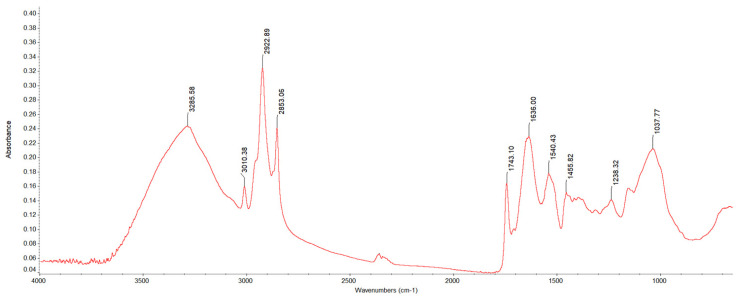
ATR-FTIR spectra of the flaxseed oilcake.

The Fourier Transform Infrared-Attenuated Total Reflection (FTIR-ATR) spectra were used to identify the key functional groups (identification of the molecular vibrations of the chemical bonds—stretching, bending, and torsions) of the macronutrients [42,80]. The FT-IR spectra showed 10 wave numbers in three different spectral regions (Figure 3). 

In the single-bond region (4000 to 2500 cm^−1^), the peak at 3285.58 cm^−1^ corresponds to the O-H stretching modes, that at 3010.38 cm^−1^ to the C-H stretching vibrations, and those at 2922.89 cm^−1^ and 2853.06 cm^−1^ to the antisymmetric and symmetric C-H stretching vibrations (mainly associated with the hydrocarbon chains found in lipids and lignins), respectively [81,82,83,84].

Three peaks characteristic of a carbonyl group and amides (I due to C=O stretching vibrations and II due to N-H bending) appear in the double-bond region at 1743.10 cm^−1^, 1636.00 cm^−1^, and 1540.43 cm^−1^ [80,85].

Polysaccharides have strong and characteristic absorption peaks in the region between 1500 and 600 cm^−1^ (fingerprint region) [83]. These peaks (1238.32 cm^−1^ and 1037.77 cm^−1^) are due to the coupling and combination of C-O stretching or C=H_2_ bending vibrational modes of individual molecular bonds [85,86,87].

### 3.4. Fatty Acid Composition of Flax Seeds and Flaxseed Oilcake

We conducted an analysis of the fatty acid profile of the oil fraction extracted from flax seeds and flaxseed oilcake, and the results are presented in Table 6. In total, 31 fatty acids were isolated and subsequently identified: 9 were classified as polyunsaturated fatty acids (PUFA), 7 as monosaturated fatty acids (MUFA), and 15 as saturated fatty acids (SFA).

The composition of flaxseed is dominated by unsaturated fatty acids (UFA, 57.40%), with a prevalence of MUFA. The following fatty acids are present in concentrations greater than 1%: pentadecanoic, palmitic, heptadecanoic, cis-10 heptadecanoic, oleic, elaidic, linoleic, and linolenic.

The most prevalent fatty acids in flaxseed oilcake are also UFAs (87.90%). Those with a proportion greater than 1% include the following: stearic, caprylic, cis-11,14-eicosadienoic erucic, pentadecanoic, cis-10-pentadecanoic, cis-8,11,14-eicosatrienoic, heptadecanoic, cis-10 heptadecanoic, oleic, linolelaidic, elaidic, linoleic, γ-linoleic, α-linolenic, nervonic, and cis-4,7,10,13,16,19-docosa-hexanoic.

Palmitoleic acid is rarely found in plants, making it difficult to introduce into the human diet. This fatty acid has been shown to reduce the risk of inflammatory diseases, diabetes, and cardiovascular diseases [88].

Excessive consumption of SFAs has been shown to have a detrimental effect on human health, particularly with regard to the cardiovascular system [89]. In this respect, the seeds were found to be richer in SFAs than the cold-pressed flaxseed oilcake.

For the human body, n-6 and n-3 PUFAs are essential because they cannot be produced and must be obtained through a balanced diet. The most essential n-3 and n-6 PUFAs are α-linolenic acid and linoleic acid, respectively. The second of these is found in seeds in lower proportions (1.65%). Among other oilseed cakes investigated in other studies, the highest percentage of linoleic acid was found in sunflower (65.97%) followed by rapeseed (39.63%) [42,53]. Flax seeds and flaxseed oilcake are the best source of omega-3 fatty acids for non-fish eaters [19].

The optimal n-6/n-3 ratio is regarded as being between 1:1 and 5:1. However, the modern diet typically exhibits a high intake of n-6 PUFAs (>10:1), which has been linked to an increased risk of developing inflammatory diseases, such as obesity [42,90]. The findings of this study indicate that the value of flaxseed oilcake (12.58) is lower that of the flax seeds (47.56). Recent studies have demonstrated that an imbalance in the n-6:n-3 PUFA ratio, with values ranging from 10 to 25, has the potential to adversely impact human health [21]. The results of the study suggest that the consumption of flaxseed oilcake may exhibit a positive nutritional profile and a beneficial effect on cardiovascular risk factors.

### 3.5. Amino Acid Composition of Flax Seeds and Flaxseed Oilcake

As shown in Table 7, the amino acid content of flaxseed oilcake was found to be higher than that of the seeds (34,814.15 nmol/g and 18,484.16 nmol/g, respectively).

In flax seeds, the most abundant amino acids (≥5%) are glutamine, glycine, alanine, and glutamic acid. Flaxseed oilcake is rich in glutamine, valine, aspartic acid, glutamic acid, asparagine, asparagine, alanine, histidine and cystine. Flax seeds and flaxseed oilcake are lower in tyrosine (respectively 2.35% and 1.28%).

The most abundant amino acid detected in both samples was glutamine, accounting for 24.37% in the flaxseed oilcake and 23.89% in the seeds. A total of three amino acids (α-aminobutyric acid, proline, and proline/hydroxyproline) were identified in the flaxseed oilcake, yet were absent from the seeds. The presence of shells has been demonstrated to have a significant impact on the digestive process. The shells serve as an intercellular barrier, which hinders the activity of digestive enzymes and, consequently, results in a reduced number of amino acids within the shells [91].

The essential amino acids under investigation in this study were valine, leucine, isoleucine, threonine, methionine, phenylalanine, histidine, lysine, and tryptophan. Specifically, leucine, isoleucine, and valine are classified as branched-chain amino acids, as they are not synthesized by the body and must thus be obtained from food sources. These amino acids serve as essential components in protein synthesis and play a crucial role in the energy metabolism [7,63]. Methionine exhibits anti-hepatitis, anti-fatty liver, and anti-cirrhotic actions, while threonine exhibits anti-anemic actions [77,92].

Threonine was absent from the seeds, while phenylalanine was present in the flaxseed oilcake. The flax seeds exhibited higher concentrations of essential AAs (76.71% and 70.46%). Histidine and valine were the predominant AAs in both samples. The relative abundance of these AAs in the flax seeds and flaxseed oilcake samples is presented in descending order, as follows: histidine, valine, methionine, tryptophan, leucine, isoleucine, isoleucine, lysine, and phenylalanine in the seeds; and valine, histidine, threonine, methionine, leucine, tryptophan, isoleucine, and lysine in the flaxseed oilcake.

Plant proteins mainly contain tryptophan, methionine, lysine, and threonine. For this reason, they are considered incomplete [77]. Flaxseed cake can be used for plant-based dairy drinks because it contains higher levels of lysine, methionine, and threonine than regular almond milk (flaxseed oilcake contains 1.45%, 1.65%, and 1.93% respectively, while almonds contain 0.7%, 0.3% and 0.7%, respectively) [93]. Other oilcakes investigated, such rapeseed (1.31%, 1.42% and 0%), sunflower (0%, 2.5% and 2.90%), sesame (3.4%, 2.3% and 3.7%), and hemp (4.16%, 1.39% and 4.57%) also contain higher values [42,53,63,94].

### 3.6. Comparison of Mineral Composition Between Flax Seeds and Flaxseed Oilcake

Minerals are defined as inorganic nutrients that are indispensable for sustaining the physico-chemical processes that define life as we know it [95]. These elements can be classified into two distinct categories: macronutrients, which include potassium, phosphorus, calcium, and sodium, and micronutrients, which encompass iron, copper, zinc, molybdenum, chromium, manganese, copper, and selenium [96].

A comparative study of mineral elements (Table 8) showed that flax seeds are the richest (17 minerals present), followed by flaxseed oilcake, with 13 minerals present. Mg was not present in the flaxseed oilcake. With the exception of Fe(III), Co, and Tl, all elements were concentrated in the flaxseed oilcake. In the literature, flax seeds are reported to contain 2.7 mg/100 g Fe, 1 mg/100 g Cu, 431 mg/100 g Mg, 3 mg/100 g Mn, and 4 mg/100 g Zn [62].

The major macro-elements identified in flax seeds were Mg and Ca, representing 70.30% of the total ash content (3.80%). In flaxseed oilcake, the only macro-element present is Ca, representing 14.53% of the total ash content (4.79%).

### 3.7. Model Fitting: Total Polyphenolic Content and Antioxidant Activity

Table 9 presents the influence of the solvent, ultrasonic time, temperature, and amplitude on the total polyphenol content (TPC) and free radical scavenging capacity by DPPH (AA_DPPH).

The application of various working parameters resulted in alterations in the polyphenol content and antioxidant activity. Consequently, TPC ranged from 3.14 mg GAE (gallic acid equivalent)/g for the sample extracted in ethanol at 40 °C for 20 min with an amplitude of 100% to 38.75 mgGAE/g for the sample extracted in methanol at 30 °C for 20 min with an amplitude of 70%.

The range of variation for the AA_DPPH method was between 27% (ethanol extraction treatment at 40˚C, 15 min at 70% amplitude) and 73.33% (methanol extraction treatment at 40˚C, 20 min at 100% amplitude). The values found are in agreement with those in the literature [97,98,99].

The results of the analysis of variance (ANOVA) (Table 10) demonstrated the adequacy of the selected mathematical models (quadratic type) for the two responses, with a *p*-value of less than 0.0001 and a capacity to explain 88% and 94% of the variance in the data.

**Figure 4:**
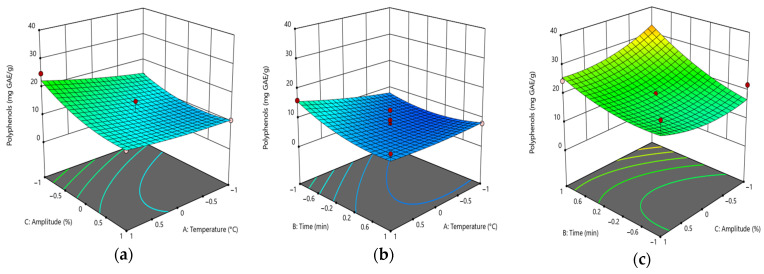

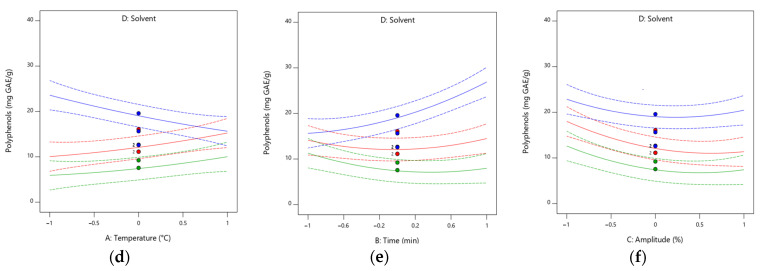
Response surfaces illustrating the effect of extraction parameters and different solvents: (**a**) water, (**b**) ethanol, (**c**) methanol. For (**d**–**f**), the blue color indicates methanol, the green color indicates ethanol, and the red color indicates water.

The total polyphenol content (TPC) was found to be the most negatively influenced by the type of solvent utilized, with amplitude also exerting a detrimental effect. The interaction between time and amplitude, as well as time and solvent type in the ethanol and water solvents, also imparts a negative influence on the response. Conversely, the interaction between temperature and solvent has a positive impact on TPC when employing water and ethanol as solvents (Figure 4a–c).

Temperature has been observed to exert a positive effect on polyphenol content when water and ethanol are employed as extraction media. Conversely, the amplitude exhibited a negative impact on the TPC response across all three solvents. The time variable demonstrated a positive effect when employing methanol followed by water as the solvent (Figure 4d–f).

The range of variation for the free radical scavenging capacity using the DPPH method ranged from 27% in the ethanol extraction treatment at 40 °C for 15 min at 70% amplitude to 73.33% in the methanol extraction treatment at 40 °C for 20 min at 100% amplitude.

**Figure 5:**
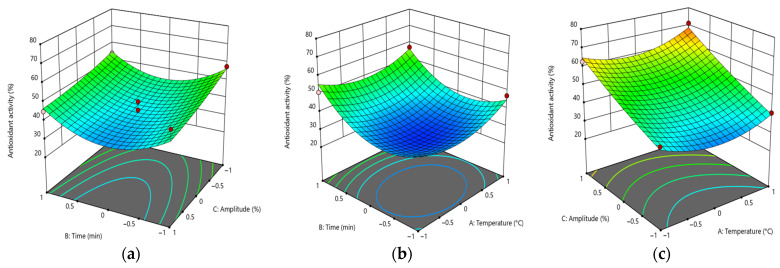

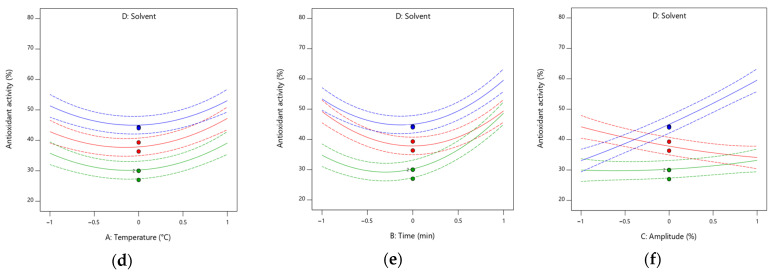
Response surfaces for the combined effects of factors on antioxidant activity when different solvents are utilized. Water (**a**), ethanol (**b**), and methanol (**c**) are used in this example. For (**d**–**f**), the blue color indicates methanol, the green color indicates ethanol, and the red color indicates water.

The analysis yielded a significant (*p* < 0.001) positive influence of linear and quadratic factors on antioxidant activity. The majority of interactions between factors exhibited a predominantly positive effect. The interaction between time and the solvent used exhibited the most significant negative influence (*p* < 0.01) for all solvents examined. Conversely, the interaction between amplitude and solvent was negative only when water and ethanol were used as the extraction media. Conversely, temperature exhibited a negative impact on the antioxidant capacity, irrespective of the solvent type. Notably, the amplitude exhibited a positive effect on the AA_DPPH value only in the context of using methanol as the solvent (Figure 5).

Comparing the methanol extracts to the ethanol extracts, the amount of total phenolics extracted by the different solvents decreased. Radical scavenging activity towards DPPH was also highest for the methanol extracts. The ethanol extracts showed the lowest radical scavenging activity towards DPPH with an intermediate total phenol content. Only the effect of single-factor time and temperature increased the phenolic content and antioxidant capacity, respectively. Other modifications of these responses depend on the synergetic effect of multiple factors.

Flaxseed oilcake polyphenols have garnered significant attention due to their inherent physiological properties, which encompass antioxidant, anti-inflammatory, anti-microbial, and anticarcinogenic characteristics [100]. These properties underscore their potential applications in various fields, including cosmetics, nutraceuticals, food production, and the fabrication of biomaterials [13]. Although flaxseed oilcakes offer potential medical effects, more research is needed to fully understand their mechanics of action and the most effective therapeutic applications [17].

This research examined the polyphenol content and antioxidant activity of flaxseed oilcake, employing a range of solvent concentrations. The most significant effects were observed when a mixture of solvents (methanol: acetone: water) was utilized, followed by 80% acetone, 80% methanol, and ethanol [97]. Barta et al. observed that a flaxseed oilcake with a particle size of less than 250 µm exhibited lower values for the two responses, indicating an uneven distribution of phenolic compounds in the seeds [67]. In a study evaluating the phenolic content of five oilcakes, the flaxseed oilcake was found to have the lowest content (3.80 mgGAE/g) [83].

### 3.8. Phenolic Acids

The extract with the highest TPC content was evaluated for the identification of individual phenolic acids. The average phenolic acid concentrations found are shown in Table 11. The phenolic acids identified in the samples occurred in the following order: vanillic acid, chlorogenic acid, caffeic acid, myricetin, quercetin, rosmarinic acid, luteolin, 4-hydroxybenzoic acid, kamferol, and p-coumaric acid. The sum of the detected phenolic compounds was 6514.97 mg/kg. This value is in line with those found in the literature (7900–84,400 mg/Kg) [13,15]. Ferulic, gallic, and sinapic acids were not present in our study, compared to the study conducted by Mueed et al. [13].

## 4. Future Directions

Flaxseed oilcake is a material that has not been adequately utilized in the fields of food science and human food systems, despite its affordability and potential benefits. Flaxseed oilcake can be used to replace animal-based protein when plant-based diets are being considered. Additionally, oilcakes can be used by consumers who choose to avoid gluten because they are naturally gluten-free.

Recognizing the potential health value of flaxseed and the rising consumer demand for healthier foods, the utilization of flaxseed oilcakes offers a promising avenue for the production of innovative products, including protein bars, fillings for patisserie products, pasta, cream, and ice cream formulas. An additional potential application lies in the development of cost-effective edible films and coatings that can compete with their oil-based counterparts.

## 5. Conclusions

The food industry is responsible for the generation of substantial amounts of waste and by-products, which represent a significant environmental concern. A considerable portion of these materials is often discarded without undergoing further utilization in secondary streams. While some materials are repurposed, a substantial amount remains underutilized or unused, despite the materials’ potential as a valuable resource.

Our comprehensive evaluation of flaxseed oilcake in relation to oilseeds resulted in the following conclusions. The process of oil removal resulted in an enhancement of nutritional value, with a substantial increase in protein content.

A residual quantity of oil is present in the oilcake. The investigation of the fatty acid composition indicates the existence of predominantly UFA. The predominant fatty acids were oleic and elaidic acids.

A further study was conducted to evaluate mineral element losses during processing. The findings revealed that, while the majority of elements exhibited an increase in content, a decrease was observed in the Fe, Co, and Li content.

Flaxseed oilcakes have high water-holding and emulsifying capacities and low foaming capacity. The emulsion stability is excellent, but the foam stability across one hour is low.

Higher temperatures and shorter ultrasound durations negatively influenced the total polyphenol content values. Increases in the ultrasound temperature, duration, and amplitude were demonstrated to exert a favorable influence on antioxidant activity.

The results of the current research corroborate the nutritional characteristics of flax seeds and flaxseed oilcake. Moreover, they emphasize the potential of flaxseed oilcake to act as a natural, valuable, and economical resource for producing of nutraceuticals and functional food products.

## Figures and Tables

**Table 1:** Experimental design for determining TPC and AA.

Run	Independent Variables							
	Coded				Actual			
	A	B	C	D	Solvent	Time, min	Amplitude, %	Temperature, °C
1	−1	−1	0	{ 1 0 }	water	10	70	30
2	1	−1	0	{ 1 0 }	water	10	70	50
3	−1	1	0	{ 1 0 }	water	20	70	30
4	1	1	0	{ 1 0 }	water	20	70	50
5	−1	0	−1	{ 1 0 }	water	15	40	30
6	1	0	−1	{ 1 0 }	water	15	40	50
7	−1	0	1	{ 1 0 }	water	15	100	30
8	1	0	1	{ 1 0 }	water	15	100	50
9	0	−1	−1	{ 1 0 }	water	10	40	40
10	0	1	−1	{ 1 0 }	water	20	40	40
11	0	−1	1	{ 1 0 }	water	10	100	40
12	0	1	1	{ 1 0 }	water	20	100	40
13	0	0	0	{ 1 0 }	water	15	70	40
14	0	0	0	{ 1 0 }	water	15	70	40
15	0	0	0	{ 1 0 }	water	15	70	40
16	−1	−1	0	{ 0 1 }	ethanol	10	70	30
17	1	−1	0	{ 0 1 }	ethanol	10	70	50
18	−1	1	0	{ 0 1 }	ethanol	20	70	30
19	1	1	0	{ 0 1 }	ethanol	20	70	50
20	−1	0	−1	{ 0 1 }	ethanol	15	40	30
21	1	0	−1	{ 0 1 }	ethanol	15	40	50
22	−1	0	1	{ 0 1 }	ethanol	15	100	30
23	1	0	1	{ 0 1 }	ethanol	15	100	50
24	0	−1	−1	{ 0 1 }	ethanol	10	40	40
25	0	1	−1	{ 0 1 }	ethanol	20	40	40
26	0	−1	1	{ 0 1 }	ethanol	10	100	40
27	0	1	1	{ 0 1 }	ethanol	20	100	40
28	0	0	0	{ 0 1 }	ethanol	15	70	40
29	0	0	0	{ 0 1 }	ethanol	15	70	40
30	0	0	0	{ 0 1 }	ethanol	15	70	40
31	−1	−1	0	{ −1 −1 }	methanol	10	70	30
32	1	−1	0	{ −1 −1 }	methanol	10	70	50
33	−1	1	0	{ −1 −1 }	methanol	20	70	30
34	1	1	0	{ −1 −1 }	methanol	20	70	50
35	−1	0	−1	{ −1 −1 }	methanol	15	40	30
36	1	0	−1	{ −1 −1 }	methanol	15	40	50
37	−1	0	1	{ −1 −1 }	methanol	15	100	30
38	1	0	1	{ −1 −1 }	methanol	15	100	50
39	0	−1	−1	{ −1 −1 }	methanol	10	40	40
40	0	1	−1	{ −1 −1 }	methanol	20	40	40
41	0	−1	1	{ −1 −1 }	methanol	10	100	40
42	0	1	1	{ −1 −1 }	methanol	20	100	40
43	0	0	0	{ −1 −1 }	methanol	15	70	40
44	0	0	0	{ −1 −1 }	methanol	15	70	40
45	0	0	0	{ −1 −1 }	methanol	15	70	40

**Table 2:** Physical properties of flax seeds (sample of 500 seeds).

Parameters	Interval	Mean Value
Size and shape		
W, mm	1.95–2.58	2.24 ± 0.20
L, mm	4.15–5.40	4.78 ± 0.21
T, mm	0.70–1.28	1.05 ± 0.08
D_e_, mm	1.81–2.47	2.27 ± 0.07
Ψ, -	0.43–0.53	0.48 ± 0.02
V, mm^3^	2.53–7.35	5.56 ± 0.59
S, mm^2^	10.23–19.06	16.15 ± 1.04
A_p_, mm^2^	6.45–10.59	8.76 ± 0.62
Gravimetric properties		
φ, %	28.34–32.36	38.97 ± 3.75
p_b_, kg/m^3^	682.88–698.39	605.19 ± 5.10
p_t_, kg/m^3^	792.22–1320.91	994.94 ± 62.40
M, g	0.0040–0.0090	0.0070 ± 0.001

W—seed width; L—seed length; T—seed thickness; φ—seed porosity; D_e_—seed equivalent diameter; Ψ—seed sphericity; M—seed mass; p_b_—bulk density of seeds; p_t_—true density of seeds.

**Table 3:** Correlations among the physical properties of flax seeds.

Variables	W	L	T	D_e_	Ψ	V	S	A_p_	M
W	**1**								
L	**0.458**	**1**							
T	0.075	−0.061	**1**						
D_e_	**0.669**	**0.595**	**0.678**	**1**					
Ψ	0.057	**−0.665**	**0.706**	**0.203**	**1**				
V	**0.609**	**0.306**	**0.825**	**0.944**	**0.502**	**1**			
S	**0.670**	**0.598**	**0.674**	**0.999**	**0.198**	**0.945**	**1**		
A_p_	**0.839**	**0.867**	0.002	**0.736**	**−0.376**	**0.526**	**0.739**	**1**	
M	**0.103**	**0.127**	**0.137**	**0.197**	0.021	**0.178**	**0.197**	**0.136**	**1**

The bold values are considered significant (*p* value < 0.05). L—seed length; W—seed width; T—seed thickness; M—seed mass; D_e_—seed equivalent diameter; Ψ—seed sphericity.

**Table 4:** Nutritive composition of flax seeds and flaxseed oilcake.

Parameter	Seeds	Oilcake
Total dietary fibers, %	26.10 ± 0.04 ^a^	24.90 ± 0.69 ^b^
Proteins, %	19.03 ± 0.01 ^b^	34.67 ± 0.17 ^a^
Ash, %	3.80 ± 0.06 ^b^	4.79 ± 0.04 ^a^
Lipids, %	27.74 ± 0.48 ^a^	11.61 ± 0.40 ^b^
Moisture, %	5.70 ± 0.02 ^b^	8.54 ± 0.11 ^a^
Remaining carbohydrates, %	36.66 ± 0.52 ^a^	15.47 ± 0.80 ^b^
Energy value, kcal/100 g	524.57 ± 2.21 ^a^	354.87 ± 2.69 ^b^

Different letters indicate significant differences among the samples (*p* < 0.05).

**Table 5:** Flaxseed oilcake characterization.

Incidence of Mycotoxins				
Properties	Limit of Detection (LOD), µg/Kg	Limit of Quantification (LOQ), µg/Kg	Results, µg/Kg	Maximum Limit 2006/576/EC, µg/Kg
Zearalenone	10	15	44.01 ± 5.08	2000
Ochratoxin A	0.5	1.5	22.19 ± 3.60	50
Aflatoxin B_1_	0.3	0.7	<LOQ	10
Deoxynivalenol	0.011	0.042	<LOD	0.9
Functional characteristics				
Oil-holding capacity (g/g)		1.19 ± 0.04		
Water-holding capacity (g/g)		4.14 ± 0.18		
Emulsion capacity (%)		26.90 ± 1.68		
Emulsion stability (%)		100.00 ± 0.00		
Foaming capacity (%)		8.41 ± 0.56		
Bulk density (g/mL)		0.6262 ± 0.001		
Least gelatinization concentration (%)		4.00 ± 0.00		
Wettability		Good: upon contact with water, the powder underwent a gradual wetting process. Some of the powder was dispersed in the water and the rest was deposited at the bottom of the Berzelius beaker. The powder settled to the bottom of the beaker after a short interval. Following a duration of half an hour, the powder particles fully settled to the bottom of the Berzelius beaker. Through the application of vortexing, the sample was dispersed throughout the liquid.		
Color parameters				
L*		52.59 ± 0.02		
a*		5.05 ± 0.03		
b*		12.72 ± 0.02		

**Table 6:** Fatty acid profile of the flax seeds and flaxseed oilcake.

Fatty Acids		Seed, %	Oilcake, %
Caprylic acid (C8:0)	SFA	0.32 ± 0.01 ^b^	1.01 ± 0.04 ^a^
Capric acid (C10:0)	SFA	-	0.37 ± 0.02 ^a^
Lauric acid (C12:0)	SFA	0.35 ± 0.01 ^b^	0.40 ± 0.01 ^a^
Tridecanoic acid (C13:0)	SFA	-	0.23 ± 0.00 ^a^
Myristic acid (C14:0)	SFA	0.46 ± 0.02 ^b^	0.61 ± 0.01 ^a^
Myristoleic acid (C14:1, n-5)	MUFA	0.19 ± 0.01 ^b^	0.31 ± 0.01 ^a^
Pentadecanoic acid (C15:0)	SFA	11.86 ± 0.04 ^a^	4.98 ± 0.14 ^b^
cis-10-pentadecanoic acid (C15:1, n-5)	MUFA	-	4.27 ± 0.04 ^a^
Palmitic acid (C16:0)	SFA	6.79 ± 0.03 ^a^	0.92 ± 0.03 ^b^
Palmitoleic acid (C16:1, n-7)	MUFA	0.19 ± 0.01 ^b^	0.68 ± 0.01 ^a^
Heptadecanoic acid (C17:0)	SFA	7.80 ± 0.14 ^a^	1.31 ± 0.07 ^b^
cis-10 heptadecanoic acid	MUFA	4.34 ± 0.10 ^b^	8.06 ± 0.16 ^a^
Stearic acid (C18:0)	SFA	1.65 ± 0.08 ^a^	1.16 ± 0.01 ^b^
Oleici acid+ elaidic acid (C18:1, cis + trans, n-9)	MUFA	20.64 ± 0.07 ^b^	34.29 ± 0.05 ^a^
Linoleic acid + Linolelaidic acid (C18:2, cis + trans, n-6)	PUFA	18.03 ± 0.19 ^b^	22.91 ± 0.16 ^a^
γ-Linolenic acid (C18:3, n-6)	PUFA	1.05 ± 0.06 ^b^	1.43 ± 0.01 ^a^
α-Linolenic acid (C18:3, n-3)	PUFA	2.46 ± 0.02 ^a^	1.65 ± 0.02 ^b^
Arachidonic acid (C20:0)	SFA	8.50 ± 0.06 ^a^	-
Gondoic acid (C20:1, n-9)	MUFA	1.90 ± 0.01 ^a^	1.08 ± 0.03 ^b^
cis-11,14-eicosadienoic acid + cis-8,11,14-eicosatrienoic acid (C20:2, n-6)	PUFA	0.43 ± 0.01 ^b^	0.87 ± 0.01 ^a^
cis-11,14,17-eicosatrienoic acid (C20:3, n-3)	PUFA	0.25 ± 0.01 ^b^	0.34 ± 0.0 ^a^
Arachidonic acid (C20:4, n-6)	PUFA	0.28 ± 0.01 ^a^	0.33 ± 0.01 ^a^
cis-5,8,11,14,17-eicosapentenoic acid (C20:5, n-3)	PUFA	0.15 ± 0.00 ^b^	0.35 ± 0.01 ^a^
Heneicosanoic acid (C21:0)	SFA	0.52 ± 0.00 ^b^	0.69 ± 0.01 ^a^
Eicosadienoic acid (C22:0)	SFA	0.15 ± 0.00 ^b^	0.23 ± 0.01 ^a^
Erucic acid (C22:1, n-9)	MUFA	4.89 ± 0.01 ^b^	5.98 ± 0.04 ^a^
cis-4,7,10,13, 16, 19-docosahexanoic acid (C22:2, n-6)	PUFA	0.18 ± 0.00 ^a^	0.30 ± 0.30 ^a^
cis-4,7,10,13,16,19-docosa-hexanoic+ nervonic acid (C22:6, n-3 + C24:1, n-9)	PUFA	2.45 ± 0.04 ^b^	5.03 ± 0.03 ^a^
Tricosanoic acid (C23:0)	SFA	4.05 ± 0.03 ^a^	-
Lignoceric acid (C24:0)	SFA	0.14 ± 0.00 ^a^	0.21 ± 0.00 ^a^
C18:2 w-6/C18:3 w-3		7.33 ± 0.17 ^b^	13.90 ± 0.09 ^a^
C18:1 w-9/C18:2 w-6		1.14 ± 0.02 ^b^	1.50 ± 0.01 ^a^
ΣSFAs (%)		42.58 ± 0.00 ^a^	12.11 ± 0.16 ^b^
ΣUFAs (%)		57.40 ± 0.26 ^b^	87.90 ± 0.25 ^a^
ΣMUFAs (%)		32.15 ± 0.02 ^b^	54.68 ± 0.09 ^a^
ΣPUFAs (%)		25.27 ± 0.24 ^b^	33.21 ± 0.16 ^a^
ΣSFAs/ΣUFAs		0.74 ± 0.003 ^a^	0.14 ± 0.002 ^b^
ΣPUFAs/ΣMUFAs		0.74 ± 0.01 ^a^	0.61 ± 0.00 ^b^

The values of different properties are expressed as the mean ± standard deviation. Mean values that are accompanied by different letters are significantly different (*p* ≤ 0.05) according to ANOVA, with Tukey’s post hoc test.

**Table 7:** Flax seeds and flaxseed oilcake: amino acid profile comparison.

Amino Acids	Seed		Oilcake	
	nmol/g	%	nmol/g	%
Glycine	1264.60 ± 14.68 ^a^	6.84	543.70 ± 0.32 ^b^	1.56
Alanine	1049.26 ± 2.66 ^a^	5.68	998.97 ± 17.55 ^b^	2.87
α-aminobutiric acid	-	-	563.09 ± 15.72 ^a^	1.62
Valine *	635.45 ± 8.47 ^b^	3.44	6140.77 ± 0.07 ^a^	17.64
Leucine *	494.13 ± 2.54 ^a^	2.67	548.76 ± 49.43 ^a^	1.58
Isoleucine *	480.28 ± 10.95 ^b^	2.60	523.94 ± 2.25 ^a^	1.50
Threonine *	-	-	673.63 ± 3.04 ^a^	1.93
Serine	657.92 ± 3.01 ^a^	3.56	674.10 ± 35.38 ^a^	1.94
Proline	-	-	571.57 ± 4.24 ^a^	1.64
Asparagine	675.17 ± 23.83 ^b^	3.65	1206.59 ± 16.49 ^a^	3.47
Thioproline	489.28 ± 0.80 ^b^	2.65	686.43 ± 5.65 ^a^	1.97
Aspartic acid	672.51 ± 22.95 ^b^	3.64	3498.83 ± 258.64 ^a^	10.05
Methionine *	533.01 ± 10.39 ^b^	2.88	573.86 ± 11.70 ^a^	1.65
3/4-Hidroxiproline	699.44 ± 9.26 ^a^	3.78	692.77 ± 30.29 ^a^	1.99
Phenylalanine *	436.78 ± 23.96 ^a^	2.36	-	-
Glutamic acid	988.98 ± 20.40 ^b^	5.35	2762.27 ± 105.61 ^a^	7.93
Glutamine	4415.02 ± 72.75 ^b^	23.89	8484.73 ± 313.81 ^a^	24.37
Ornithine	543.93 ± 0.57 ^a^	2.94	566.32 ± 26.46 ^a^	1.63
Glycylproline	447.08 ± 3.25 ^a^	2.42	460.41 ± 0.52 ^a^	1.32
Hidroxylysine	518.35 ± 0.12 ^a^	2.80	553.12 ± 42.19 ^a^	1.59
Proline-Hydroxyproline	-	-	453.36 ± 10.67 ^a^	1.30
Histidine *	719.46 ± 12.55 ^a^	3.89	786.66 ± 0.16 ^a^	2.26
Lysine *	475.92 ± 7.50 ^b^	2.57	503.29 ± 0.69 ^a^	1.45
Tyrosine	434.65 ± 0.34 ^a^	2.35	446.57 ± 14.88 ^a^	1.28
Tryptophan *	530.36 ± 3.60 ^a^	2.87	532.56 ± 1.85 ^a^	1.53
Cystathionine	656.81 ± 0.18 ^a^	3.55	663.97 ± 9.10 ^a^	1.91
Cystine	665.76 ± 14.77 ^a^	3.60	703.88 ± 0.12 ^a^	2.02
Total AA	18,484.16		34,814.15	
Essential AA %	76.71		70.46	
Non essential AA %	23.29		29.54	

The values are expressed as the mean ± standard deviation. The presence of different superscript letters (a, b) indicates that the values are statistically different at the 95% confidence level (ANOVA, Tukey’s post hoc test). *: essential amino acids.

**Table 8:** Flax seeds and flaxseed oilcake: mineral comparison.

Mineral Elements	Seed	Oilcake
Beryllium (Be), mg/Kg	20.50 ± 0.50 ^b^	38.90 ± 1.90 ^a^
Lithium (Li), mg/Kg	2.20 ± 0.10 ^a^	2.10 ± 0.10 ^a^
Molybdenum (Mo), mg/Kg	0.30 ± 0.00 ^a^	0.50 ± 0.02 ^a^
Magnesium (Mg), mg/Kg	4694.90 ± 290.50 ^a^	-
Calcium (Ca), mg/Kg	974.40 ± 90.00 ^b^	2006.20 ± 112.00 ^a^
Titan (Ti), mg/Kg	12.00 ± 0.70 ^b^	23.50 ± 1.20 ^a^
Chromium (Cr), mg/Kg	71.50 ± 6.10 ^b^	147.00 ± 4.50 ^a^
Manganese (Mn), mg/Kg	63.80 ± 3.30 ^b^	173.40 ± 2.20 ^a^
Cesium (Ce), mg/Kg	359.30 ± 27.00 ^b^	9025.50 ± 763.00 ^a^
Iron (Fe-II), mg/Kg	4.50 ± 0.30 ^b^	7.60 ± 0.20 ^a^
Iron (Fe-III), mg/Kg	2.40 ± 0.59 ^a^	2.10 ± 0.10 ^a^
Cobalt (Co), mg/Kg	6.10 ± 0.10 ^a^	2.60 ± 0.10 ^b^
Nickel (Ni), mg/Kg	18.30 ± 1.70 ^b^	26.50 ± 1.10 ^a^
Copper (Cu), mg/Kg	50.40 ± 3.30 ^b^	82.60 ± 2.90 ^a^
Zinc (Zn), mg/Kg	74.70 ± 4.70 ^b^	107.60 ± 4.10 ^a^
Selenium (Se), mg/Kg	596.40 ± 13.10 ^b^	1319.60 ± 33.40 ^a^
Thallium (Tl), mg/Kg	1111.9 ± 65.00 ^a^	839.40 ± 68.10 ^a^
Total, mg/Kg	8063.60	13,805.10

Different superscripts letters (a, b) after the values indicated differences statistically significant at *p* < 0.05% (ANOVA, Tukey test).

**Table 9:** Experimental values reported for TPC and AA_DPPH for the realized extracts.

Parameters		Run	TPC, mg GAE/g	AA_DPPH, %
Temperature (°C)	30	1	11.55	50.07
		7	10.43	36.70
		35	25.00	42.00
	40	9	16.57	57.62
		11	16.00	50.56
		12	11.09	44.96
		40	31.94	44.87
		44	15.64	44.33
	50	17	16.04	47.16
		23	8.65	44.96
		38	17.00	71.07
Time (min)	10	24	13.27	33.59
		39	22.00	44.32
		41	24.49	70.71
	15	13	11.13	44.04
		14	16.09	36.35
		21	13.15	37.89
		29	7.56	27.00
		43	12.59	44.25
	30	18	8.00	51.01
		25	14.00	46.97
		42	24.48	73.33
Amplitude (%)	40	10	22.00	53.92
		20	11.43	39.31
		36	22.62	40.71
	70	2	16.99	57.54
		3	14.00	57.62
		15	11.13	39.31
		16	6.90	43.15
		31	15.22	55.00
		34	22.60	65.60
	100	8	13.20	39.00
		22	6.06	40.31
		37	26.00	62.61
Solvent	Water	4	15.53	60.03
		5	13.82	47.37
		6	24.88	52.63
	Ethanol	19	11.00	57.19
		26	13.16	30.47
		27	3.14	54.75
		28	9.23	30.00
		30	12.67	30.00
	Methanol	32	11.00	60.61
		33	38.75	71.66
		45	19.59	44.00

**Table 10:** Regression coefficients and the evaluation of mathematical models for TPC and AA_DPPH, *** *p* < 0.001; ** *p* < 0.01; * *p* < 0.05.

Variables	TPC	DPPH
R^2^	0.8823	0.9433
Adjusted R^2^	0.8082	0.9076
F value	11.91	26.43
*p* value	<0.0001	<0.0001
Lack of Fit	0.5130	0.1361
Constant	+12.85	+37.70
A	+0.23	+1.57 *
B	+1.39 *	+3.38 ***
C	−2.37 ***	+3.26 ***
D1	−0.76 ***	+0.15 ***
D2	−5.44 ***	−7.45 ***
A·B	−1.83 *	−1.21
A·C	−1.17	+1.07
A·D1	+2.37 ***	+0.6138
A·D2	+1.83 ***	+0.1117
B·C	−2.59 **	+0.9228
B·D1	−1.20 ***	−3.29 **
B·D2	−3.04 ***	+3.56 **
C·D1	−0.9446	−8.30 ***
C·D2	−0.2308	−1.67 ***
A^2^	+0.5628	+7.20 ***
B^2^	+2.22 *	+11.49 ***
C^2^	+2.61 **	+1.32

**Table 11:** Phenolic compounds identified in the flaxseed oilcake.

Phenolic Acids	Results, mg/Kg
4-Hydroxybenzoic acid	17.22 ± 0.68
Vanillic acid	2599.00 ± 149.18
Caffeic acid	62.51 ± 1.37
Chlorogenic acid	2415.81 ± 235.41
p-cumaric acid	14.71 ± 1.54
Rosmarinic acid	61.14 ± 5.46
Myricetin	968.39 ± 32.95
Luteolin	24.68 ± 2.21
Quercetin	335.02 ± 8.98
Kaempferol	16.49 ± 0.54
Total	6514.97

## Data Availability

The original contributions presented in the study are included in the article; further inquiries can be directed to the corresponding authors.
